# Living with complexity; marshalling resources: a systematic review and qualitative meta-synthesis of lived experience of mental and physical multimorbidity

**DOI:** 10.1186/s12875-015-0345-3

**Published:** 2015-11-24

**Authors:** Peter A. Coventry, Nicola Small, Maria Panagioti, Isabel Adeyemi, Penny Bee

**Affiliations:** Collaboration for Leadership in Applied Health Research and Care Greater Manchester and Manchester Academic Health Science Centre, University of Manchester, Manchester, UK; NIHR School for Primary Care Research and Manchester Academic Health Science Centre, University of Manchester, Manchester, UK; School of Nursing, Midwifery and Social Work and Manchester Academic Health Science Centre, University of Manchester, Manchester, UK

**Keywords:** Multimorbidity, Meta-synthesis, Self-management

## Abstract

**Background:**

Multimorbidity poses a major health burden worldwide yet most healthcare is still orientated towards the management of single diseases. Literature on the experience of living with multimorbidity is accumulating but has not yet been synthesised in a manner conducive to informing the design of self-management interventions for this population. This study aimed to systematically review and synthesise findings from published, in-depth qualitative studies about the experience of multimorbidity, with a view to identifying the components and motivation for successful self-management in this population.

**Methods:**

Systematic review of and meta-synthesis of qualitative studies that evaluated patient experience of living with and/or self-managing mental and/or physical multimorbidity. MEDLINE, Embase, PsycINFO, CINAHL, and ASSIA along with reference lists of existing reviews and content pages of non-indexed specialists comorbidity journals were searched.

**Results:**

Nineteen studies from 23 papers were included. A line of argument synthesis was articulated around three third-order constructs: 1) Encounters with complexity; 2) Marshalling medicines, emotions, and resources; and 3) Self-preservation and prevention. Our interpretation revealed how mental and physical multimorbidity is experienced as moments of complexity rather than mere counts of illnesses. Successful self-management of physical symptoms was contingent upon the tactical use of medicines, whilst emotional health was more commonly managed by engaging in behavioural strategies, commonly with a social or spiritual component. Motivations for self-management were underpinned by a sense of moral purpose to take responsibility for their health, but also by a desire to live a purposeful life beyond an immediate context of multimorbidity.

**Conclusions:**

Understanding how people experience the complexities of mental and physical multimorbidity may be crucial to designing and delivering interventions to support successful self-management in this population. Future self-management interventions should aim to support patients to exert responsibility and autonomy for medical self-management and promote agency and self-determination to lead purposeful lives via improved access to appropriate social and psychological support.

**Electronic supplementary material:**

The online version of this article (doi:10.1186/s12875-015-0345-3) contains supplementary material, which is available to authorized users.

## Background

Multimorbidity occurs in the majority of patients with long term conditions, and contributes substantially to health inequalities [1, 2]. Multimorbidity reduces quality of life, and increases mortality, primary care consultations [1] and unplanned hospital admissions [3, 4]; mental illness and economic deprivation can exacerbate these problems. A recent nationally representative study of 1.75 million people in Scotland showed that multimorbidity occurred 10–15 years younger in deprived areas, with multimorbidity that included both mental and physical health problems being over twice as common in the most deprived localities [2]. Similar patterns have been reported in English general practice surveys [5].

Self-management has been defined by the UK Department of Health as “…the care taken by individuals towards their own health and well-being: it comprises the actions they take to lead a healthy lifestyle; to meet their social, emotional, psychological and physical needs; to care for their long-term condition; and to prevent further illness or accidents” [6]. Support for self-management is critical to the delivery of effective care for people with long term conditions, but achieving this is a challenge in multimorbid populations [7]. Primary care is often too fragmented and lacks continuity for people with multimorbidity, leading to inconvenience and hassles when interacting with multiple components of health services [8]. For their part practitioners report task uncertainty, burn out and heart sink from the ‘endless’ struggle to coordinate and manage the care of people with multimorbidity, making the delivery of patient centred care for this population less realisable [9, 10].

The lived experience of multimorbidity is likely to be critical to self-management. A narrative review of predictors of self-management decision making and priority setting found that perceptions and attitudes about the importance of a particular ‘dominant’ illness partially drove self-management practices in patients with multimorbidity [11], but that importantly, the designation of their dominant illness was not static and could change from day-to-day depending on severity of symptoms, impacts, long term consequences and available support and treatments. This review focused exclusively on the practical aspects of self-management however, making it difficult to identify generalisable and meta-level themes about how the experience of the emotional as well as the physical consequences of multimorbidity might link to self-management. Quantitative studies of self-management behaviours in patients with combined mental and physical multimorbidity are available but are limited in their ability to elucidate the more nuanced meanings of living with multiple and potentially conflicting long term conditions.

There is a growing need to give greater prominence to the patient experience of multimorbidity to better understand how to design patient (and family) centred interventions for this population. This philosophy is underscored by more mature expressions of patient or user involvement in health services research as conceived by approaches modelled on experienced based co-design [12] and user-focused monitoring [13]. As a first step towards this goal it is critical to first identify the existing evidence base and develop appropriate theory about the rationale for interventions [14], for example by using secondary analysis such as evidence synthesis. This study therefore aims to use meta-synthesis to synthesise findings of qualitative studies about living with and coping with mental and physical multimorbidity.

## Methods

We conducted a systematic search of qualitative studies and synthesised the data from included studies using meta-ethnographic approaches. These approaches, originally devised by Noblit and Hare [15] have been previously adapted for utility in the syntheses of qualitative data in healthcare research [16, 17]. Our study had three phases: 1. systematic search of qualitative literature; 2. data extraction; and 3. translation of second order constructs and line of argument synthesis. This review is reported in accordance with the PRISMA checklist (Additional file 1).

### Search strategy

We searched MEDLINE, Embase, PsycINFO, CINAHL, and ASSIA: Applied Social Sciences Index and Abstracts from inception to April 2015. The searches used MeSH and free text words organised in into three blocks: 1. multimorbidity; 2. qualitative designs; and 3. patient/user experience. A comprehensive list of search terms and search results per electronic database are shown in Additional file 2. Search terms were derived from existing reviews of multimorbidity [18] and reviews of qualitative health research [19] along with input from PB whose previous work includes searches of qualitative studies as part of health technology assessments [20]. We supplemented the electronic search by checking references of existing reviews about multimorbidity and checked the content of journals not indexed by the electronic databases searched, but which specialise in publishing research about multimorbidity (Journal of Comorbidity; SAGE Open Medicine).

### Inclusion and exclusion criteria and study screening

Table 1 lists the inclusion and exclusion criteria. To be included studies had to meet minimum quality criteria: only studies as defined by the British Sociological Association criteria for evaluating qualitative research papers were included [21]. Our research goal was to explore patient experience of living with and coping with multimorbidity regardless of the types or combination of illnesses, reflecting the fact that multimorbidity is often characterised by uncertainty and a state of flux, and self-management priorities can change from day to day [22]. With this in mind we opted to use a definition of multimorbidity that captures the presence of two or more chronic illnesses (more commonly known as long term conditions) where no one condition is more important than another [23]. This contrasts with the concept of comorbidity which is typically used to define an index condition along with one or more comorbid conditions which may affect the course and treatment of the index condition [24]. Following the practice of previous reviewers, we excluded studies that reported exclusively about comorbidity on the grounds that these studies dealt with a conceptually different category of phenomena to those that focus on multimorbidity [18].

**Table 1:** Study inclusion and exclusion criteria

Inclusion criteria	Exclusion criteria
Peer reviewed journal articles or conference papers about primary research, published in English.	Unpublished papers, dissertations, book chapters.
Used a qualitative design, defined as those studies that collect data using specific qualitative techniques such as unstructured interviews, semi-structured interviews or focus groups, either as a stand-alone methodology or as discrete part of a larger mixed-method study, and analysed qualitatively.	Studies that collected data using qualitative methods but then analysed these data using quantitative methods.
Participants with physical and/or mental multimorbidity, defined as “the co-existence of two or more chronic conditions, where one is not necessarily more central than the others” (ref Valderas).	Studies that included participants with <2 long term conditions; had a diagnosis of severe mental health problems (e.g. psychosis), substance/alcohol abuse (i.e. dual diagnosis), cancer, terminal illness, or in receipt of palliative care.
Studies that reported patients’ experiences of living with multimorbidity and/or self management of multimorbidity.	Studies that reported health care professionals' experiences of addressing multimorbidity; described patients experiences of specialist care services (e.g. cancer services); described patients’ experiences of interventions designed to support self-management.

Beyond the need for included studies to report primary qualitative data (collected and analysed using methods described in Table 1) our broad inclusion criteria focused on studies that reported data about the patient experience of living with and coping with multimorbidity. Studies that included only health professional narratives or combined data from patient and health professional participants were excluded in-keeping with the rationale that greater understanding of the day-to-day lived experience of multimorbidity is likely to explain barriers and success to self-managing multimorbidity.

All records from electronic searches were imported into EndNote and duplicates removed. Titles and abstracts were then imported to Covidence (www.covidence.org), a web based platform to aid screening and maintenance of systematic reviews. Titles and abstracts were independently screened by 5 reviewers against review inclusion criteria. This initial process excluded 4716 reports; 82 full text reports were retrieved. Full text reports were split between two pairs of reviewers (PC and NS; MP and IA) and assessed against the broad inclusion criteria. Judgements about inclusion were made blind in each pairing of reviewers and disagreements resolved by discussion in each pairing; unresolved disagreements were resolved at a consensus meeting of all reviewers.

**Fig. 1:**
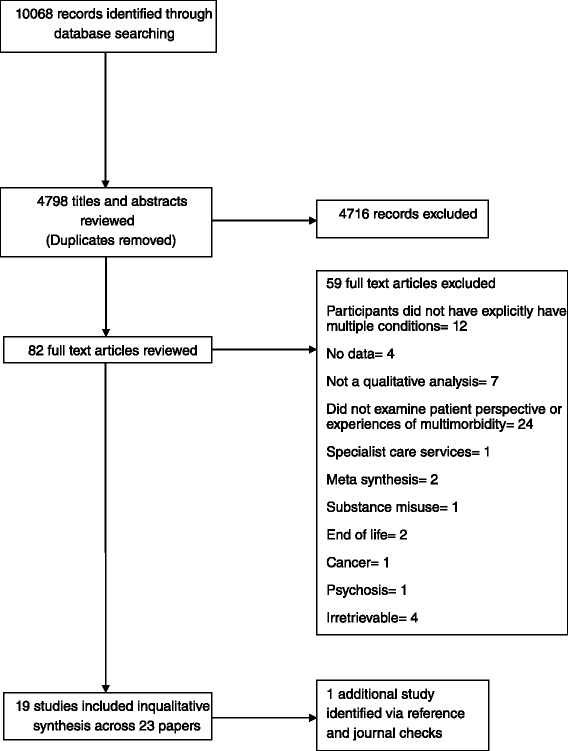
PRISMA flowchart

As Daker-White et al. have argued, in meta-synthesis this latter phase of screening full text papers is an inductive and iterative process that yields both quantitatively and qualitatively different results than systematic reviews of effectiveness, where the goal is often to maximise the amount of data retrieved to enhance the power of meta-analyses [25]. While we did draw up a pre-specified set of inclusion and exclusion criteria modelled on the PICO formula the process of screening full texts demanded that we implement a more fine grained set of exclusion criteria to 1) limit the number of studies that could be feasibly synthesised using meta-ethnography and 2) maximise homogeneity and ‘fit’ between studies, thereby enhancing our chances of conducting a line of argument synthesis. During consensus discussions we therefore agreed to exclude studies that focused on cancer and end of life experiences as the narratives included in these studies spoke to a different (and often therapeutic) agenda beyond support for self-management. Similarly we excluded studies that focused on patient experience of substance misuse or so-called dual diagnosis, and severe and enduring mental illness. People with these conditions are typically in receipt of specialist mental health care and self-management interventions for these groups are less likely to translate to the broader population with mental and physical multimorbidity. Following these principles we finally included 19 studies reported across 23 papers in the synthesis. Figure 1 shows the study flow using the PRISMA flowchart.

### Data extraction and synthesis

Descriptive data about study aims and country of origin, number of long term conditions at recruitment, data collection and analysis methods, and sample characteristics (size, age, number/type of long term conditions, sex, ethnicity, deprivation and setting) were extracted by one reviewer (NS) using a form adapted from a published meta-ethnography [26].

Meta-ethnography is a systematic but interpretative approach to analysis that begins with noting verbatim and coded text in terms of first-order and second-order constructs, translation of constructs across papers in the synthesis to form third-order constructs, and finally synthesis using either reciprocal, refutational, or line of argument approaches [15]. When referring to first-, second and third-order constructs, we defined these in the same way as they are defined by Britten et al. [27]. First-order constructs represent the primary data reported in each paper; second-order constructs are defined as the authors’ (often metaphorical) interpretations of the primary data; and third-order constructs are the reviewers’ interpretations derived from a tertiary analysis of the first and second-order constructs.

In our study full text reports were evenly split between three reviewers (PC, NS, MP) who independently re-read these reports and extracted first and second order constructs. This process involved mapping primary data against second order constructs and verbally reporting these findings at a meeting devoted to cross-tabulating first and second order constructs across all studies until we arrived at a comprehensive but loosely organized table of findings. This process alerted the review team to the fact that the studies could be broken down into three subsets with a focus on: 1) the physical, psychological and social impact of multimorbidity; 2) self-management practices and coping strategies; and 3) motivations and reasoning for self-management. Some studies included first and second order constructs that cut across these categories whereas others had a more narrow focus. The analysis team revisited the full text papers and (re)allocated first and second order constructs under these broad thematic headings. These thematic headings were not treated as third-order constructs, rather they served as a way to signpost the wealth of first and second-order constructs that addressed our core research questions. This process was iterative and each version of the table of first and second-order constructs was shared between the reviewers who cross checked their version derived from their set of papers against equivalent versions from team members. This back and forth process was analogous to the process of constant comparison in primary research i.e. translating findings from each study into one another until agreement was reached about how to link first and second-order constructs to third order interpretations [17, 28]. Because there was overlap but also differences between studies we opted to narratively synthesize findings using a line of argument that related to three core third order constructs.

## Results

Additional file 3: Table S2 shows the key characteristics of the included studies. Ten studies were conducted in the United States [15, 29–37]; six studies in the United Kingdom [38–45], and one in Canada [11, 12], Amsterdam [26], and in Germany [35].

Multimorbidity was measured in 18 studies by counting the number of conditions participants had at time of recruitment; 11 studies recruited participants with at least 2 or more long-term conditions [7, 13, 26, 31, 34, 36, 37, 40, 45–47]; four studies recruited participants with at least ≥3 long term conditions [11, 12, 14, 20, 35]; one study recruited participants with ≥ 4 conditions [38, 43, 44, 48], and another study ≥5 conditions [24]. One study measured multimorbidity by grouping high users of medical services [49].

Thirteen studies presented counts or frequencies of the different types of chronic conditions patients had to manage; hypertension was the most commonly reported type of condition in four studies [30, 31, 35, 36]; high blood pressure in three studies [33, 34]; depression in three studies [37, 41, 45]; diabetes in two studies [29, 39]; and arthritis in one study [47, 50].

Across the studies the sample sizes ranged from seven to one hundred participants (depending on method of data collection). Age ranged from 30 to 96 years; ten studies recruited middle aged (40+) to elderly participants (80+ years): [32–38, 41–44, 48, 49]; the majority of studies, except two [42, 51], recruited predominately more women.

Eight studies did not report ethnicity [31, 36, 38, 39, 41–44, 46, 49, 52, 53]. In six studies, the majority of participants were White participants [30, 31, 40, 47, 50, 51], and Black participants [32–34]; two studies recruited predominantly participants from black and ethnic minority communities [35, 37].

Eleven studies recruited from a primary and community care setting [29, 31–34, 36, 38–40, 42–44, 47–50]; eight studies recruited from secondary care [30, 34, 35, 37, 41, 45, 51, 53]. Eleven studies recruited a relatively homogenous socioeconomic population [29, 31, 33, 36, 40, 42–45, 47–51, 53]; seven studies recruited primarily patients living in areas of high deprivation [32, 34–37, 39, 41]; and one study recruited an affluent population [30].

Data were collected in 13 studies by semi-structured interview [30, 32, 36–45, 47–51]; five studies used focus groups [13, 23, 34, 37, 41]; and one study used a combination of both methods [47]. Data were analysed in eight studies thematically [29, 32, 34, 36, 40, 42, 47, 50, 51]; five studies used the constant comparative method [30, 31, 36, 41, 43, 44, 48, 49]; two studies followed established phenomenological procedures [37, 45]; two studies used a framework analysis method [35, 39]; and two studies conducted a basic content analysis [32, 53].

### Data synthesis

Our analysis of first order data across second order themes resulted in a line of argument organised around three inter-related third order constructs:

- 2^nd^ order: Characterising the lived experience of multimorbidity → 3^rd^ order: ‘Encounters with complexity’
- 2^nd^ order: Self-managing multimorbidity → 3^rd^ order: ‘Marshalling medicines, relations, and emotions’
- 2^nd^ order: Motivations and meanings of self-management of multimorbidity → 3^rd^ order: ‘Self-preservation and prevention’

#### Encounters with complexity

Additional file 4: Table S3 includes the findings that were translated into one another to support this third order interpretation of the experience of multimorbidity. A predominant theme across many of the studies related to descriptions about the bodily and emotional impact of multimorbidity. In these studies first order data were organised around second order constructs that pointed up the corporeal reality of living with multiple chronic illnesses over time, with an emphasis on functional loss and impairment. This was highlighted by data that illustrated the way in which multimorbidity had imposed severe restrictions on mobility, more so than with the effects of single conditions [37, 44, 45, 48, 50], suggesting that the bodily effects of multimorbidity as a whole may be greater than that of the individual long term conditions [39]. The disabling consequences of multimorbidity were underscored by a sense in which bodies had become broken, had begun to fall apart and grind to a halt, leading to much more circumscribed and less rewarding lives [36, 37, 42, 44, 45, 48, 49].

With impairment comes chronic fatigue, sometimes induced by sleeplessness (owing to pain or medication side effects [37, 45]), but other data also showed that fatigue was associated with living with and dealing with multimorbidity, with second order constructs suggestive of an embattled existence with no let up from the disabling consequences of illness [41]. The demands of living with multimorbidity are such that every day can be a struggle, exhausting people’s capacity to complete every day tasks. ‘Getting through the day in one piece’ was a refrain that cut across many studies that focused on how the illness work associated with managing multimorbidity could leave people without any energy to live a life beyond their illnesses. Existential crises about the loss of active and productive lives and negativity about a life restricted to just ‘doing’ multimorbidity were prevalent themes that brought together data about the physical and emotional consequences of multimorbidity [36, 41, 42, 44, 45]. For some the bodily and emotional affects of multimorbidity damaged relations with their family and partners, thus destroying the social fabric of their lifeworlds too [36, 37, 48].

These themes and data highlight that multimorbidity is more than the sum of its parts. Indeed, as O’Brien et al. found, the concept of ‘multimorbidity’ was rarely voiced by patients across studies and bears little relation to the broad range of physical, social and emotional experience summoned by the presence of multimorbidity [41]. Here we would argue that multimorbidity is better characterised as an encounter with complexity, dealing as it does with the impact of illness on both bodily and emotional health and attendant social consequences. In this sense multimorbidity is less about a collection of illnesses but is often experienced as a complex state oscillating between existing (getting through the day) and non-existence, (running down time on a life) in “…a place where they send you to die really,” [41] with the thought that “I might not be able to do anything here anymore…” [49] and “. . . Life is just not the same anymore” [48].

#### Marshalling medicines, relations, and emotions

Additional file 5: Table S4 includes the findings that were translated into one another to support this third order interpretation of the content and practices of self-management in multimorbidity. Owing to the wide reaching effects of multimorbidity first order data about self-care were organised around second order themes that offered insight into a broad range of medical and behavioural strategies about managing the bodily, emotional, and social consequences of multimorbidity.

In keeping with what we know about self-managing single long term conditions decision making about medicine(s) taking (on the part of the patient) also assumed a central place in the self management of multimorbidity [46]. Medical self-management of multimorbidity revolved around amelioration of physical symptoms (e.g. pain and fatigue), but because of the multi-faceted nature of these symptoms the data included under this theme highlighted how participants’ decision making about medicines taking was not straightforward. Complexity here was not solely about the number of medicines that needed to be taken (although this was an issue for some) [39], rather it stemmed from the need to find a balance between competing outcomes [31]: taking medicines to control symptoms while minimising the risk of side effects. Here the data fractured between two modes of medicine taking. On the one hand a major theme in this data found that people sometimes adopted disciplined and regimented approaches to taking either over the counter and/or prescribed medicines for managing symptoms, especially pain [32, 35, 36, 43, 51]. The emphasis here was being in control and introducing regularity and routine into an otherwise uncertain daily schedule. Whereas in other data, and predominantly so, people adopted a more flexible approach that involved making tactical decisions about which medicines to take [30, 39, 43], at what dose [34, 47, 49] and, in countries without social medicine, at what financial cost [30, 33]. Both approaches channelled a discourse around patients being strategic and marshalling medicines taking to their best advantage.

Whereas drugs played a central role in managing physical symptoms there was (apart from one instance – [44]) great resistance to using them to manage the emotional consequences of multimorbidity. In part this reluctance to engage in medical management of mood stemmed from a broader reluctance to rely only on medicines for self-management [30, 43], but also from fear of taking anti-depressants and other drugs owing to risk of antagonistic side-effects (e.g. [40, 49]). More typically resistance to taking anti-depressants was better explained in the context of confidence with and enthusiasm for using behavioural strategies and alternative therapies to control mood problems. Marshalling emotions was variously achieved by changing the pace and place of every day life, by undertaking exercise (inside or outside) and taking recreational trips outdoors [32, 33, 44, 45]. Related but not equivalent to this data were findings that showed how for some people with multimorbidity, alternative medicines and therapies held sway over conventional approaches to managing mood, chiefly yoga, meditation and herbal medicines [33, 42]. An important strand of data also showed how doing everyday tasks such as house work (and perhaps less commonly, cleaning the church) played a crucial role in managing emotional distress [32, 41, 49]. Everyday house work, instead of paid work, can offer a distraction from the emotional pain of multimorbidity. In the absence of such distractions there is a risk that mental health can worsen, leading to rumination and existential crises: “Sometimes I sit and cry. I do. I sit and cry ‘cause I think ‘God’s sake you’re only 50 years of age. How did it come to this that you’re in so much pain…I can’t make sense of it” [41].

The final component of this third order construct centres on people’s capacity to marshal relations between themselves and significant others (including God) to self-manage the impact of multimorbidity. The data that speaks to this element split three ways between seeking companionship and supporting others, invoking social comparisons with others, and the value of prayer and spirituality. Receiving or giving social support was typically characterised as a buffer against the stresses of living with multimorbidity, either by co-opting family members in helping them get through the day [37, 42, 49, 53], helping and enabling others [33], or serving as a distraction from emotional distress [35, 44, 48, 50]. Similarly, social comparison (with others who were more unwell) was a tactic employed to distance those with multimorbidity from the stresses and strains of their own lives [32, 33, 35]. In the absence of social support a minority of people with multimorbidity from faith based communities in the Unites States drew on daily prayer and maintenance of their relationship with God to gain strength and succour to get through the day [32, 33, 49].

#### Self-preservation and prevention

Additional file 6: Table S5 includes the findings that were translated into one another to support this third order interpretation of the motivations and meanings of self-management in multimorbidity. The final third order construct is derived from analysis of data organised under second order themes that pertained to deciphering and understanding the motivations for and meaning of self-management in multimorbidity. These interpretative themes cut across the descriptive themes about the content and process of self-management elaborated on under the previous third order construct. However, owing to their interpretative rather than descriptive content, data were treated as analytically separate and synthesised under a new third order construct about preserving function and self-identity and prevention of future decline.

Findings around preservation of function link back to findings about reactive self-management to control and ameliorate symptoms. Self-monitoring or ‘body listening’ [33, 49] and ‘doctoring themselves’ [34, 35] were typical second order themes that invoked a sense in which medicines taking was undertaken in accordance with well understood theories about self-regulation [54]. But in other ways, because participants across studies rarely had a coherent sense of what multimorbidity is, tactical decisions about medical self-management were often also informed by a more instrumental and proactive formula based on priority setting, the limits of what constituted acceptable levels of medicine taking, and the desire to preserve and fulfil social roles [29, 30, 39, 43, 44].

We discussed in the section above that many people across multiple studies and multiple settings chose to manage the emotional impact of multimorbidity by recourse to behavioural and social coping strategies. Beyond a dislike of medicines a major theme that underscored findings about motivations for using these approaches centred on preservation of the self and self-identity. In the presence of physical and emotional hardship a significant strand of data spoke to people’s motivation to draw on a moral impetus to stay strong and disciplined and true to themselves [33, 41, 47] and prevent further decline by declaring an intention to “…go ahead and do it. It’s a case of me fighting the disease” [48]. In addition to a belief that inner strength to battle on in the face of hardship was synonymous with moral rectitude this stoic approach was also driven by people’s desire to remain independent. This was illustrated by the perception that asking for help or using aids (e.g. sticks or chair lifts) might dent self-identities built on being autonomous and able to carry on ‘as normal’ under difficult circumstances [35, 41, 44].

Adjacent to this theme about retaining independence was a smaller but important strand of data that stood apart from this narrative about preserving self identity via disciplined stoicism and instead pointed to how for some, being purposeful and enacting a sense of agency formed the basis of their self-management practices. This was seen vey much in studies that reported how people’s behavioural self-management strategies sat outside the regularities of managing the physical components of multimorbidity and were instead focused on ensuring that they invested in autonomous activities that preserved a future as well as a present self, thereby enhancing a state of well-being [33, 35, 37, 45].

## Discussion

Whole system approaches that integrate mental and physical healthcare and support self-management for people with multimorbidity are called for, but few primary and community based interventions to do this currently exist [55]. This systematic review and meta-synthesis was undertaken on the grounds that a broader understanding of patient experience of living with and coping with mental and physical multimorbidity is likely to be critical to designing interventions that support self-management in this population. Our interpretation of the evidence about how the experience of multimorbidity relates to self-management of multimorbidity led to a line of argument that conceived multimorbidity as a state of complexity that is often strategically self-managed by marshalling medical and behavioural resources to preserve self-identity and prevent further decline.

While there is evidence that people with three or more conditions experience poorer quality of life than those with one or two conditions [5], our findings suggest that the phenomenology of multimorbidity is experienced as a complex state that goes beyond counts of conditions and symptom burden and incorporates psycho-social problems played out against a backdrop of uncertainty and constant flux. This can in part be explained by revisiting existing and well understood notions about biographical disruption and flow that relate to how multimorbidity (as with single long term conditions) can invoke a sense of repeated and anticipated continuity and discontinuity owing to fluctuations in physical and emotional function [56, 57]. But it also highlights that as with the presentation of mental illness in primary care [58], multimorbidity is often experienced in complex and undifferentiated forms, with overlap between physical and emotional symptoms and temporal variation over the life course. Additionally the physical and emotional hardships were most keenly felt among those in remote rural and heavily deprived urban areas highlighting how the social and environmental context can shape the experience of multimorbidity. In this sense multimorbidity is very much about moments of complexity and our findings showed that how these are experienced and made sense of has some significant implications for self-management.

Our findings showed that across all types of multimorbidity and all age groups self-management of physical symptoms was heavily reliant on medicines taking. Furthermore, the majority of the studies that contributed to this element of our interpretation highlighted that medicines taking was often enacted in a strategic way to maximise the chances of achieving a balance between benefits and side-effects. In-keeping with the findings of Bratzke et al. [11] decision making here was often grounded on instrumental rationality [59], in that people with multimorbidity commonly made calculated judgements about whether to either take medicines or not take them, and to take them at different doses and at different times dependent on priorities and context. In some cases this mode of medicines taking was expressed as tacit knowledge or ‘know how’ accrued from years of experience of self-managing often antagonistic symptoms and competing goals, reinforcing the benefits of recalibrating relationships between patients and health care professionals in favour of an empowered model that supports patients to draw on experiential learning to self-manage multimorbidity [60].

However the reach of medical self-management was limited in the context of the emotional consequences of multimorbidity. Our interpretation of the findings about self-management of depression showed that a large cross section of people with multimorbidity have a deep sense of responsibility to marshal all available non-medical resources to cope with the emotional consequences of living with multimorbidity. As understood from previous work in the sociology of chronic illness [61], multimorbidity was experienced as a moral opportunity to preserve self and reinstate agency by living out opportunities that escaped the temporal and spatial bounds of multimorbidity, for example, by enacting social roles outside the home or enabling others. Perhaps of critical importance here is the need for a better understanding of what can support agency and purposeful action in the context of multimorbidity. Hitlin and Johnson have elaborated on a novel construction of agency that proposes that agency in the modern life course needs to incorporate an understanding about perceived capacities and perceived life chances [62]. The latter is linked to the idea that an optimistic sense of life expectations (what we will become) is contingent in part on a future time perspective [63] which is often diminished in people with multimorbidity. Perceived capacity, in the form of both material and social resources, has also been shown to be critical to successful self-management in multimorbidity [64], but is a scarce resource in areas of both rural and urban deprivation leading to worsening emotional health in this population [65].

### Strengths and limitations

The validity of our search strategy was checked against a set of known papers on multimorbidity. The initial round of title and abstract searches were not double screened which may have reduced opportunities to compare and discuss how the broad inclusion criteria were applied, leading to less reliable results. However we did double screen a random selection of 100 titles and abstracts and inter-rater reliability was sufficiently good to move forward with single screening the remainder of the search results. All full text papers retrieved were double screened in pairs blind to each others ratings.

No formal critical appraisal exercise was undertaken. Quality assessments in qualitative reviews are controversial and their value is debatable [66]. Our interpretative efforts to translate findings into one another to produce third order constructs necessarily relied more heavily on studies that included richer or ‘thicker’ data. In this sense ‘the doing of the synthesis’ approximated to critical appraisal based on our interpretative judgements about the ability of each study to contribute to the synthesis rather than on any preconceived and formulaic notion of study quality. This approach has been used by one of the authors (PB) in previous qualitative reviews [20] and has been successfully deployed in a previous meta-synthesis [19].

By synthesising data across multiple papers drawn from studies conducted in different settings with different sub-groups of patients (e.g. older versus younger adults, white versus black and ethnic minorities) we may have ironed out subtle but important differences in findings in favour of a line of argument that sought out similarities. This approach may have potentially reduced the external validity of our synthesis. However we take the view that to conduct a meta-synthesis using line of argument approaches inevitably results in some loss of specificity and is less able to accommodate high levels of heterogeneity between findings than say a synthesis that draws on a refutational approach. Despite this the method of meta-synthesis that we used is still capable of drawing out conflicting cases, as we did, for example, between findings around the positive use of anti-depressant medication versus findings about the negative perceptions of anti-depressant medication. In a sense this debate is akin to the ‘lumping’ or ‘splitting’ debate in meta-analysis where there is a strong argument to always meta-analyse, even in the presence of high levels of heterogeneity [67]. We would argue the same is partly true for meta-synthesis because in the absence of third order interpretations the end product is one step removed from a narrative overview of individual studies.

### Implications for intervention design

Designing effective interventions that integrate mental and physical healthcare for people with multimorbidity is a key challenge for health systems with ageing populations [68]. The findings that people with multimorbidity resist taking medicines to manage mood problems is in-keeping with the broader understanding that people prefer non-drug therapies for depression [69]. In the UK, the National Institute for Health and Care Excellence recommend the use of collaborative care for managing depression and anxiety with long term conditions [70]. The evidence base for collaborative care is very solid. Collaborative care is more effective than usual care for managing depression and anxiety over the short, medium, and long term [71, 72]. Most evidence is derived from the US, including those trials that have shown positive effects for collaborative care for people with depression and long term conditions [73]. However the CADET trial showed that the benefits can translate to the NHS [74] and the COINCIDE trial recently showed that collaborative care can benefit people with mental-physical multimorbidity from deprived backgrounds: it can improve depression and lead to better self-management [52].

However engagement in collaborative models is variable. General practitioners might not fully sign up to the collaborative model [75], and it might be hard to ‘sell’ this model of care to people with multimorbidity from deprived backgrounds, suggesting that there is need to develop alternative ways to either deliver psychological therapy and/or include social interventions too [76]. How social resources can be optimised via care delivered in primary care is a huge challenge. Interventions delivered in partnership with community based assets are increasingly seen as a way to maximise the health benefits of existing social resources [77], but their effectiveness and indeed availability for people with multimorbidity is not known. Our findings suggest that in areas of high deprivation and few social resources innovations such as multi-disciplinary health and social care groups modelled on the framework outlined by Goodwin et al. [78] may be best placed to signpost and refer patients with multimorbidity to such community groups.

Our interpretation that people with agency and a future orientation had a more successful approach to self-management points towards testing the effectiveness of care models that support patients to make self-care plans that reflect their priorities. The Chronic Care Model proposes that support for self-management is contingent on knowledgeable, confident, and activated patients, prepared and informed clinical teams, and responsive and flexible organisational processes [79]. Care planning via House of Care has been successfully implemented in diabetes [80], and may be an effective way to support self care of people with multimorbidity. Collaborative goal setting and action planning are core features of this approach and if embedded into routine primary care of multimorbidity can potentially activate and empower people to sustain healthy self-management behaviours [81].

## Conclusions

This meta-synthesis included a diverse range of studies that qualitatively evaluated the experience and meaning of living with and coping with the mental as well as the physical components of multimorbidity. Translating findings from these individual studies into a collective whole allowed us to develop a line of argument synthesis that showed that mental and physical multimorbidity is experienced in ways that go beyond counts of illnesses. Instead the day to day experience of multimorbidity can be broken down into moments of complexity, with physical symptoms typically managed by the tactical use of medicines, whereas emotional health is more commonly managed by adopting and adapting behavioural strategies. A desire to preserve self-identity and reclaim a future were critical drivers of successful self-management, signalling a place for interventions that promote agency and self-determination, not least in areas of deprivation where social and economic resources are scarce and the affects of mental and physical multimorbidity are experienced in their most acute form.

## Additional files

Additional file 1:
**PRISMA 2009 checklist.** (DOC 62 kb) Additional file 2:
**Search Results.** (DOCX 40 kb) Additional file 3: Table S2. Characteristics of included studies. (DOCX 40 kb) Additional file 4: Table S3. Translational table of findings about encounters with complexity. (DOCX 16 kb) Additional file 5: TableS4. Translational table of findings about marshalling medicines, relations and emotions. (DOCX 24 kb) Additional file 6: Table S5. Translational table of findings about (self)-preservation and prevention. (DOCX 17 kb)
