# Supplementary material for: Living with complexity; marshalling resources: a systematic review and qualitative meta-synthesis of lived experience of mental and physical multimorbidity
Source: BMC Fam Pract. 2015 Nov 24;16:171. doi: 10.1186/s12875-015-0345-3 (PMC4657350; doi:10.1186/s12875-015-0345-3)
Supplement: Additional file 2: — Search Results. (DOCX 40 kb) [file 12875_2015_345_MOESM2_ESM.docx]

**Additional file 2: Search Results**

| **Database** | **Search run**  **9 April**  **Saved** |
| --- | --- |
| Medline search  Limits:  English language  Humans  Journal article | Multiple morbidit$.ab,ti. **169**  Multimorbid$.ab,ti. **1217**  multi-morbidit$.ab,ti. **142**  -------  (multiple adj (diseases OR illnesses OR conditions OR disorders)).ab,ti. **1539**  --------  ((co-occurrence or cooccurence or co-occurring or cooccuring or concurrent) adj2 (disease$ or illness$ or condition$ or disorder$)).ab,ti. **4104**  (multiple$ adj5 (chronic disease$ or chronic illness$ or chronic condition$)).ab,ti. **1009**  cumulative illness.ab,ti. **281**  polypathy.ab,ti. 25  polypathology.ab,ti. 48  polypathologies.ab,ti. 10  polymorbid$.ab,ti. 198  multiple pathologies.ab,ti. 217  multi$patholog$.ab,ti. 12  -----  (long$term OR long term adj (diseases or illnesses or conditions or disorders)).ab,ti. **751**  ------  (chronic adj2 (diseases or illnesses or conditions or disorders)).ab,ti. **56204** |
| Total number of hits: | **64362** |
| Qualitative studies | grounded theory.ab,ti. **5702**  interview$.ab,ti. **215706**  content analysis.ab,ti. **11097**  focus group$.ab,ti. **21025**  discourse$.ab,ti. **8555**  ethnograph$.ab,ti. **5748**  ethnonursing.ab,ti. 87  phenomenological$.ab,ti. **8922**  qualitative$.ab,ti. **142842** |
| Total number of hits | 355516 |
| User experience | Experience$. ab, ti. **671686**  Perspective$. ab, ti. 167410  View$. ab, ti. 313374  Feedback. ab, ti. 77842  Barrier$. ab, ti. 149997  Facilitator$. ab, ti. 12531  Perceiv$. ab, ti. **120482**  Perception$. ab, ti. 142140  Impact$. ab, ti. 534917  Preference$. ab, ti. 88972  Acceptability. ab, ti. 17391  Coping. ab, ti. 32499  Patient$. ab, ti. 4461328  service user$. ab, ti. 2310  Service$user$. ab, ti. 1  User$. ab, ti. **106217**  Consumer$. ab, ti. **39555**  Client$. ab, ti. **37801**  Survivor$. ab, ti. **62771**  Attitude$. ab, ti. 93123  treatment adj2 burden. ab, ti. 700  Challenge$. ab, ti. 340913  Implication$. ab, ti. 374126  Meaning$. ab, ti. 74974  Consequence$. ab, ti. 269154  Identit$. ab, ti. 99873  Opinion$. ab, ti. 60920  Report$. ab, ti. 2414394  Life Change Event$. ab, ti. 93  Life style. ab, ti. 7751  Lifestyle. ab, ti. 456358  Belief$. ab, ti. 48769  Satisfaction$. ab, ti. 73847  Collaboration$. ab, ti. 39978 |
|  | **7835508** |
| all terms together: | **3751**  **Limited to humans, English language and journal article: 3397** |
|  |  |
| Embase  Limits: human  English language  Journal article | Multiple morbidit$.ab,ti. **267**  Multimorbid$.ab,ti. **2116**  multi-morbidit$.ab,ti. **295**  -------  (multiple adj (diseases OR illnesses OR conditions OR disorders)).ab,ti. **2255**  --------  ((co-occurrence or cooccurence or co-occurring or cooccuring or concurrent) adj2 (disease$ or illness$ or condition$ or disorder$)).ab,ti. **5717**  (multiple$ adj5 (chronic disease$ or chronic illness$ or chronic condition$)).ab,ti. **1564**  cumulative illness.ab,ti. **517**  polypathy.ab,ti. 29  polypathology.ab,ti. 128  polypathologies.ab,ti. 18  polymorbid$.ab,ti. 334  multiple pathologies.ab,ti. 342  multi$patholog$.ab,ti. 24  -----  (long$term OR long term adj (diseases or illnesses or conditions or disorders)).ab,ti. **1112**  ------  (chronic adj2 (diseases or illnesses or conditions or disorders)).ab,ti. **82683** |
| Total: | **95054** |
|  | grounded theory.ab,ti. **7739**  interview$.ab,ti. **289074**  content analysis.ab,ti. **14930**  focus group$.ab,ti. **29533**  discourse$.ab,ti. **11164**  ethnograph$.ab,ti. **7200**  ethnonursing.ab,ti. 90  phenomenological$.ab,ti. **12444**  qualitative$.ab,ti. **196372** |
| Total: | **478832** |
|  | Experience$. ab, ti. 981730  Perspective$. ab, ti. **233931**  View$. ab, ti. **434375**  Feedback. ab, ti. **107080**  Barrier$. ab, ti. **208477**  Facilitator$. ab, ti. **16743**  Perceiv$. ab, ti. **162767**  Perception$. ab, ti. **191868**  Impact$. ab, ti. **829103**  Preference$. ab, ti. **117526**  Acceptability. ab, ti. **25096**  Coping. ab, ti. **46603**  Patient$. ab, ti. **6471646**  service user$. ab, ti. **3745**  Service$user$. ab, ti. 6  User$. ab, ti. **147932**  Consumer$. ab, ti. **54607**  Client$. ab, ti. **51420**  Survivor$. ab, ti. **89449**  Attitude$. ab, ti. **126765**  treatment adj2 burden. ab, ti. **1361**  Challenge$. ab, ti. **468173**  Implication$. ab, ti. **486559**  Meaning$. ab, ti. **108662**  Consequence$. ab, ti. **365106**  Identit$. ab, ti. **118904**  Opinion$. ab, ti. **90531**  Report$. ab, ti. **3337585**  Life Change Event$. ab, ti. 101  Life style. ab, ti. **12309**  Lifestyle. ab, ti. **71027**  Belief$. ab, ti. **65853**  Satisfaction$. ab, ti. **110945**  Collaboration$. ab, ti. **64182** |
| Total: | **10818480** |
| all terms together: | **5707**  **Limited to: humans, English language, article: 3443** |
|  |  |
| PsycInfo  Limits: peer reviewed journal  Human  English language | Multiple morbidit$.ab,ti. **48**  Multimorbid$.ab,ti. **318**  multi-morbidit$.ab,ti. **53**  -------  (multiple adj (diseases OR illnesses OR conditions OR disorders)).ab,ti. **374**  --------  ((co-occurrence or cooccurence or co-occurring or cooccuring or concurrent) adj2 (disease$ or illness$ or condition$ or disorder$)).ab,ti. **2453**  (multiple$ adj5 (chronic disease$ or chronic illness$ or chronic condition$)).ab,ti. **391**  cumulative illness.ab,ti. **134**  polypathy.ab,ti. 0  polypathology.ab,ti. 12  polypathologies.ab,ti. 3  polymorbid$.ab,ti. 6  multiple pathologies.ab,ti. 33  multi$patholog$.ab,ti. 2  -----  (long$term OR long term adj (diseases or illnesses or conditions or disorders)).ab,ti. **303**  ------  (chronic adj2 (diseases or illnesses or conditions or disorders)).ab,ti. **12118** |
| Total: | **15628** |
|  | grounded theory.ab,ti. **10217**  interview$.ab,ti. **233438**  content analysis.ab,ti. **14981**  focus group$.ab,ti. **21862**  discourse$.ab,ti. **38488**  ethnograph$.ab,ti. **19199**  ethnonursing.ab,ti. 46  phenomenological$.ab,ti. **19951**  qualitative$.ab,ti. **108146** |
| Total: | **362515** |
|  | Experience$. ab, ti. 451877  Perspective$. ab, ti. **196780**  View$. ab, ti. **239023**  Feedback. ab, ti. **47072**  Barrier$. ab, ti. **43439**  Facilitator$. ab, ti. **9201**  Perceiv$. ab, ti. **173993**  Perception$. ab, ti. **217715**  Impact$. ab, ti. **236553**  Preference$. ab, ti. **73252**  Acceptability. ab, ti. **7986**  Coping. ab, ti. **58384**  Patient$. ab, ti. **531387**  service user$. ab, ti. **3846**  Service$user$. ab, ti. 0  User$. ab, ti. **58323**  Consumer$. ab, ti. **38397**  Client$. ab, ti. **79803**  Survivor$. ab, ti. **20455**  Attitude$. ab, ti. **163840**  treatment adj2 burden. ab, ti. **139**  Challenge$. ab, ti. **134822**  Implication$. ab, ti. **289046**  Meaning$. ab, ti. **122795**  Consequence$. ab, ti. **105373**  Identit$. ab, ti. **85223**  Opinion$. ab, ti. **37578**  Report$. ab, ti. **532648**  Life Change Event$. ab, ti. **141**  Life style. ab, ti. **2546**  Lifestyle. ab, ti. **16343**  Belief$. ab, ti. **94229**  Satisfaction$. ab, ti. **78333**  Collaboration$. ab, ti. **27597** |
| Total: | **2332506** |
| all terms together: | **2161**  **Human, English language and peer reviewed journal, journal article: 1612** |
|  |  |
| CINAHL  (EBSCO platform)  **AB and TI restrictions**  Limits: English language  Research article/ journal article  Peer reviewed | "Multiple morbidit*" **56**  Multimorbid* **468**  multi-morbidit* **63**  -------  "multiple diseases" 91  "multiple illnesses" 30  "multiple conditions" 102  "multiple disorders" 54  --------  "chronic diseases" 4503  "chronic health diseases" 2  "chronic medical diseases" 12  "chronic illnesses" 1549  "chronic health illnesses" 1  "chronic medical illnesses" 38  "chronic conditions" 3287  "chronic health conditions" 671  "chronic medical conditions" 492  ------  "cumulative illness" **101**  polypathy 0  polypathology 9  polypathologies 0  polymorbid* 13  "multiple pathologies" 34  multi*patholog* 3  -----  "Long term diseases" 20  "long term illnesses" 45  "long term conditions" 712  "long term disorders" 8  ------ |
| total | **11623** |
|  | "grounded theory" **6302**  interview* **98961**  "content analysis" **9318**  "focus group*" **15513**  discourse* **6318**  ethnograph* **4988**  ethnonursing 157  phenomenological* **6917**  qualitative* 55,787 |
| Total: | 145,937 |
|  | Experience* **186442**  Perspective* **70336**  View* **62702**  Feedback **14122**  Barrier* **35900**  Facilitator* **4911**  Perceiv* **54851**  Perception* **59665**  Impact* **132549**  Preference* **16459**  Acceptability **5388**  Coping **20294**  Patient* **758097**  service user* **4271**  User* **35089**  Consumer* **16336**  Client* **29516**  Survivor* **19422**  Attitude* **42101**  treatment burden 632  Challenge* **83788**  Implication* **95492**  Meaning* **28632**  Consequence* **34651**  Identit* 12144  Opinion* **17141**  Report* **333573**  Life Change Event* **73**  Life style **1328**  Lifestyle **17987**  Belief* **24586**  Satisfaction* **36690**  Collaboration* **22520** |
| Total: | 1,435,278 |
| all terms together: | 1,494  English Language; Peer Reviewed; Research Article; Publication Type: Journal Article: 1,097 |
|  |  |
| Applied Social Sciences Index and Abstracts (ASSIA)  Limits: peer reviewed | "Multiple morbidit*" **19**  Multimorbid* **77**  multi-morbidit* **13**  -------  "multiple diseases" 12  "multiple illnesses" 11  "multiple conditions" 25  "multiple disorders" 19  --------  "chronic diseases" 922  "chronic health diseases" 0  "chronic medical diseases" 3  "chronic illnesses" 439  "chronic health illnesses" 0  "chronic medical illnesses" 13  "chronic conditions" 853  "chronic health conditions" 175  "chronic medical conditions" 139  ------  "cumulative illness" **27**  polypathy 0  polypathology 4  polypathologies 3  polymorbid* 1  "multiple pathologies" 1  multi*patholog* 0  -----  "Long term diseases" 6  "long term illnesses" 7  "long term conditions" 115  "long term disorders" 6  ------ |
| total | 2677 |
|  | "grounded theory" **2630** interview* 45913 "content analysis" **3575**  "focus group*" **7223**  discourse* **6248**  ethnograph* **3780**  ethnonursing 6  phenomenological* **2552**  qualitative* **22843** |
| total | 70535 |
|  | Experience* **67410**  Perspective* **23838**  View* **26058**  Feedback **5084**  Barrier* **10408**  Facilitator* **1860**  Perceiv* **29160**  Perception* **26766**  Impact* **40448**  Preference* **8307**  Acceptability **1973**  Coping **9759**  Patient* **75284**  service user* **3838**  User* **11746**  Consumer* **6112**  Client* **12871**  Survivor* **3710**  Attitude* **22396**  treatment burden 1110  Challenge* **22303**  Implication* **45833**  Meaning* **14009**  Consequence* **14127**  Identit* 12630  Opinion* **4875**  Report* **91585**  Life Change Event* **672**  Life style **1065**  Lifestyle **4526**  Belief* **15011**  Satisfaction* **13580**  Collaboration* **5203** |
| Total: |  |
| all terms together: | 582 Limit: peer reviewed: 560 |
