# Supplementary material for: Living with complexity; marshalling resources: a systematic review and qualitative meta-synthesis of lived experience of mental and physical multimorbidity
Source: BMC Fam Pract. 2015 Nov 24;16:171. doi: 10.1186/s12875-015-0345-3 (PMC4657350; doi:10.1186/s12875-015-0345-3)
Supplement: Additional file 3: Table S2. — Characteristics of included studies. (DOCX 40 kb) [file 12875_2015_345_MOESM3_ESM.docx]

**Additional file 3:Table S2: Characteristics of included studies**

| Study (First author, year, country) | Aim | Number of long-term conditions at recruitment | Data collection (IV, FG, PO) | Qualitative methodological approach | Sample (type of long-term condition (number), size, age, sex, ethnicity, location, deprivation) |
| --- | --- | --- | --- | --- | --- |
| Noel, 2005, United States | To explore the collaborative care needs and preferences in  primary care patients with multiple chronic illnesses | - At least 2+ conditions | FG | Basic thematic analysis | - Various multiple conditions [type/ number not reported]; - 60 Patients, 30 – 80 years, 48 men; - Majority of participants were White, also African-Americans and Hispanics; - 8 Geographically dispersed primary care clinics within the Veterans Health Administration, 4 clinics were in large urban metropolitan settings, 4 clinics were located in rural communities with populations of 60 000 or less; - 4 Primary care clinics were tertiary care Hospitals, 4 freestanding community-based outpatient clinics |
| Clark, 2013,2013, Western Canada | To examine self-care as a means of managing the physical realities  of having multiple chronic conditions in later life | - At least 3-14 conditions, at least one of which was arthritis, back problems, cataracts/glaucoma, diabetes, or heart disease | In-depth, semi-structured IV | Thematic analysis aided by constant comparative method | - Arthritis (28); back problems (26); heart disease (24); cataracts/glaucoma (14); and cancer (12); - 35 patients, 73-91 yrs (men mean age 78.6 yrs, women men age 80.3 yrs), - recruited from the greater Vancouver regional district, a large urban centre; - Diverse with respect to incomes, educational attainment, work histories, and countries of origin |

**Table 2: Characteristics of included studies (Continued)**

| Study (First author, year, country) | Aim | Number of long-term conditions at recruitment | Data collection (IV, FG, PO) | Qualitative methodological approach | Sample (type of long-term condition (number), size, age, sex, ethnicity, location, deprivation) |
| --- | --- | --- | --- | --- | --- |
| Coventry, 2014, United Kingdom | How does mental-physical multimorbidity express itself in lived time  and space? A phenomenological analysis of encounters with depression and chronic physical illness | - At least 3 conditions: COPD, anxiety and depression | IV | Phenomenological | - Severe COPD (11), moderate COPD (10), very severe COPD (1), rheumatoid arthritis (4), heart disease (3), or type 1 (1) diabetes, type 2 (2) diabetes, anxiety (32) and depression (32); - 32 Patients, 30-82 years, 15 men; - One of three acute hospitals in Greater Manchester who were referred to a nurse-led early respiratory discharge scheme |
| Bower, 2012, United Kingdom | To examine  patients’ representations of multimorbid long-term conditions and sought  to assess how models of illness representation might need modification in  the presence of multimorbidity. | - At least 2 of 5 conditions: noninsulin-dependent diabetes mellitus, osteoarthritis,   COPD, coronary heart disease (CHD) or depression | Semi-structured IV | Framework analysis | - Various multiple conditions such as cancer, thyroid disease and hypertension [number not reported], plus diabetes (23), COPD (12), chronic arthritis (12), CHD (11), depression (9); - 28 Patients, 39-89 years, 16 men - Patients were registered with six general practices in Greater Manchester which were part of the comprehensive local research network |

**Table 2: Characteristics of included studies (Continued)**

| Study (First author, year, country) | Aim | Number of long-term conditions at recruitment | Data collection (IV, FG, PO) | Qualitative methodological approach | Sample (type of long-term condition (number), size, age, sex, ethnicity, location, deprivation) |
| --- | --- | --- | --- | --- | --- |
| Grundberg, 2014, Amsterdam | To describe the experiences of health-promoting dialogues from the perspective of community-dwelling seniors with multimorbidity | - At least 2+ conditions: mental health conditions and other conditions [not reported] | In-depth, semi-structured IV | Content analysis | - Various multiple conditions [type not reported] sampled from geriatric clinics for elderly people with MM; - 7 Elderly, widowed, patients; 83-96 years; 1 man - All patients lived in their own homes in a suburb of Stockholm |
| Mishra, 2011, United States | To explore facilitators and barriers to adherence to multiple  medications among low-income patients with comorbid chronic physical and mental health  conditions | - At least 2+ conditions | FG | Thematic analysis | - High blood pressure (33), high cholesterol (25), depression (21), diabetes (20), chronic back pain (20), arthritis (18), asthma (11), acid reflux (10), chronic bronchitis/emphysema (9), heart disease (8), osteoporosis (8), headache/migraine (6), obesity (6), incontinence (5), nerve pain (4); - 50 Patients, 40-60+ years, 20 men - 8 White; 42 Black; - University Family Medicine (UFM), an urban outpatient clinic of the University of Maryland, Baltimore (UMB) School of   Medicine, Department of Family and Community Medicine;   - Low-income |

**Table 2: Characteristics of included studies (Continued)**

| Study (First author, year, country) | Aim | Number of long-term conditions at recruitment | Data collection (IV, FG, PO) | Qualitative methodological approach | Sample (type of long-term condition (number/frequency), size, age, sex, ethnicity, location, deprivation) |
| --- | --- | --- | --- | --- | --- |
| Loeb, 2006, United States | To identify strategies used by community dwelling African American elders to cope with their chronic  health conditions | - At least 2+ conditions | FG | Content analysis | - Blood pressure (14), vision problems (12), arthritis (11), diabetes (6), high cholesterol (5), sinus trouble (5), urinary or bladder problems (5), circulation problem (3), foot problem (3), lung problem (3), thyroid problem (3), depression (2), gout (2), heart problem (2); - 28 Patients, 55-89 years, female (69%) - African American elders; - Participants were recruited through events, such as picnics, grocery day, bingo, and support groups offered by two urban senior centers in the state of Delaware |
| Schoenberg, 2011, United States | To improve our  understanding of the ways in which vulnerable, rural residents in the United States experience and manage MM | - At least 2+ conditions | Semi-structured IV | Thematic analysis aided by constant comparative method | - Average of four chronic, self-reported conditions, including heart disease or hypertension (90%), arthritis (80%), type 2 diabetes (75%), cancer (10%), stroke (10%) - 20 Patients, 41-55+ years, women (85%) - Rural Appalachian residents; recruited from a federally qualified health clinic in Appalachian, Kentucky - Poverty rate is twice that of the nation as a whole, the per capita income is slightly over half the U.S. average |

**Table 2: Characteristics of included studies (Continued)**

| Study (First author, year, country) | Aim | Number of long-term conditions at recruitment | Data collection (IV, FG, PO) | Qualitative methodological approach | Sample (type of long-term condition (number), size, age, sex, ethnicity, location, deprivation) |
| --- | --- | --- | --- | --- | --- |
| Sells, 2009, United States | To describe and better understand adults’ responses to the onset, accrual and influence of multiple chronic conditions and to social support in adapting to consequent difficulties | - Multiple conditions, defined as ‘high users of medical services’ | Semi-structured IV | Phenomenological | - Various multiple conditions such as: chronic obstructive lung disease, sarcoidosis, hypertension, aortic   insufficiency, hypothyroidism, near total blindness [number not reported], psychiatric diagnoses (19): depression (13);   - 33 Patients, mean age 50 years, 10 men; - Largely racial/ethnic minority groups; - Random sample from an urban primary care clinic, the Yale-New Haven Hospital Primary Care Centre; - Low income, urban population residing in close proximity to the clinic |
| Loffler, 2012, Germany | To analyse the way patients aged 65–85 cope with multimorbidity | - At least 3 conditions, including at least one musculoskeletal   disorder, and those patients who experienced a certain level of health related  limitations and restrictions | Narrative in-depth IV | Grounded theory, constant comparative method | - Various multiple conditions such as: hypertension, coronary heart disease, congestive heart failure, diabetes mellitus, type 2, COPD, arterial fibrillation, coxarthrosis, gonarthrosis, lumbar spinal stenosis, and major depression [no numbers reported]; - 19 Patients, 65-85 years (mean age 75 years), 9 men; - Hamburg and several urban and rural areas within North Rhine-Westphalia; - Homogeneous socio-economic status |

**Table 2: Characteristics of included studies (Continued)**

| Study (First author, year(s), country) | Aim | Number of long-term conditions at recruitment | Data collection (IV, FG, PO) | Qualitative methodological approach | Sample (type of long-term condition (number), size, age, sex, ethnicity, location, deprivation) |
| --- | --- | --- | --- | --- | --- |
| Ridgeway, 2014, United States | To report on factors that patients with multimorbidity draw on to lessen perceptions of treatment burden. | - At least 2+ conditions: diabetes and heart failure; - involved in a complex regimen of self-care, including taking multiple medications, monitoring health status, and/or following diet and exercise plans recommended by a health care provider | IV, FG | Framework analysis | - Gastrointestinal problems (15), hypertension (28), arthritis/joint pain (21), diabetes (19), cardiovascular disease (13), depression or anxiety (21), hyperlipidemia (8), back/neck problems (8), eye problems (8), sleeping problems (9) [IV/FG combined data]; range 1-16; - 100 Patients, 47-87 years, mean age 60 years; male (50%) - More minorities represented (89%) - IV patients recruited through flyers posted in the waiting area of the primary care outpatient clinic;   FG participants were recruited through patient advisory groups and disease-specific treatment clinics.   - Data collected at Mayo Clinic, Rochester, Minnesota and at Hennepin County Medical Center (HCMC), Minneapolis, Minnesota. HCMC is a hospital and ambulatory clinic system providing care for low-income |

**Table 2: Characteristics of included studies (Continued)**

| Study (First author, year(s), country) | Aim | Number of long-term conditions at recruitment | Data collection (IV, FG, PO) | Qualitative methodological approach | Sample (type of long-term condition (number), size, age, sex, ethnicity, location, deprivation) |
| --- | --- | --- | --- | --- | --- |
| Townsend, 2003,2006,2008,2011, Scotland | To examine attitudes towards drug use among middle aged respondents with high levels of chronic morbidity. | - At least 4+ conditions | Semi-structured IV | Constant comparative method, narrative | - Various multiple conditions such as: disc injury, kidney problems, arthritis, hypertension, asthma, diabetes, osteoporosis, blood clots, anxiety, depression, irritable bowel syndrome, sinus problems, sciatica, ovarian cysts, migraine, tinnitus, migraine, hypertension, inner ear problems, breast cancer, anxiety, depression, panic attacks, gastric ulcer, colitis, duodenal ulcer, thyroid   Problems, eczema [number not reported];   - 23 patients, early 50s, 10 men; - recruited from a community health survey in Scotland; - West of Scotland - Equal numbers of people renting and home owners |

**Table 2: Characteristics of included studies (Continued)**

| Study (First author, year(s), country) | Aim | Number of long-term conditions at recruitment | Data collection (IV, FG, PO) | Qualitative methodological approach | Sample (type of long-term condition (number), size, age, sex, ethnicity, location, deprivation) |
| --- | --- | --- | --- | --- | --- |
| Simmonds, 2013, United Kingdom | To explore (i) primary care (PC) patients’  perceptions of links between their physical condition and mental health, (ii) their experiences of living with  depression and CHD and (iii) their own self-help strategies and attitudes to current PC interventions for depression | - At least 2 conditions: depression and coronary heart disease | Semi-structured IV | Basic thematic analysis, aided by constant comparative method | - Various multiple conditions such as: depression, coronary heart disease, diabetes, high blood pressure, asthma, COPD, obesity, thyroid problems [number not reported]; - 30 Patients, 47-85 years, - 30% African/Afro-Caribbean - Participants were drawn from primary care registers from 4 South London Boroughs (Lambeth, Lewisham, Southwark and Croydon) coterminous with Primary Care Trusts (PCTs) in South East and South West London; - Ethnically diverse population |

**Table 2: Characteristics of included studies (Continued)**

| Study (First author, year(s), country) | Aim | Number of long-term conditions at recruitment | Data collection (IV, FG, PO) | Qualitative methodological approach | Sample (type of long-term condition (number), size, age, sex, ethnicity, location, deprivation) |
| --- | --- | --- | --- | --- | --- |
| Leach, 2008, United States | To increase our  understanding of how older adults attempt to manage MM | - At least 2+ conditions | In-depth, open ended and semi structured IV | Basic thematic analysis | - Various multiple conditions such as, high blood pressure (38), arthritis (32), diabetes (18), cancer (11), other diseases (14), stroke (4); - 41 Patients, 55-84 years mean age of 70 years, 6 men ; 44% widowed; - 63% Black ethnicity (self-described); - All participants were from an urban or suburban context in Kentucky |
| Corser, 2011, United States | To explore the perceived health care needs of adults with numerous comorbid conditions by focusing on their self-management  practices and relationships with primary care providers | - At least 2 conditions: chronic cardiac, pulmonary, or endocrine   health conditions | FG | Basic thematic analysis | - Greater than 50% diabetes (type 1 or type 2), chronic pulmonary disease (i.e., asthma, chronic obstructive pulmonary disease, chronic emphysema), congestive heart failure, coronary artery disease; other conditions included osteoarthritis or musculoskeletal disorder, and/or ongoing cancer/neoplasm [number not reported]: four+ (18) conditions; - 18 Patients, 34-83 years, 4 men - 3 African or other minority; - Midwestern academic-based family medicine clinic |

**Table 2: Characteristics of included studies (Continued)**

| Study (First author, year(s), country) | Aim | Number of long-term conditions at recruitment | Data collection (IV, FG, PO) | Qualitative methodological approach | Sample (type of long-term condition (number), size, age, sex, ethnicity, location, deprivation) |
| --- | --- | --- | --- | --- | --- |
| Elliot, 2007, United States | To explore how older adults with multiple illnesses make choices about medicines. | - At least 3 conditions: those considered   ‘symptomatic’ (such as GI disease, arthritis) versus ‘asymptomatic’ (such as hypertension, hypercholesterolemia) and mental health medication | Semi-structured IV | Constant comparative method | - Hypertension (15); hypercholesterolemia (15); ischemic heart disease (5); heart failure (3); thromboembolic disease (2); mitral valve disease (1); arrhythmia (1); GI disease (7); pain (6); neurological pain (4); arthritis (3); leg cramps (2); gout (1); migraine (1); diabetes mellitus (6);   osteoporosis (5); glaucoma (3); macular degeneration (3); anxiety (5); depression (2); ADD (1); cancer undergoing treatment (3); allergies (2); psoriasis/eczema (2); COPD; range 3-9 conditions;   - 20 Patients, 67-90 years, 8 men - 2 African American; - Community dwelling older adults from Harvard Pilgrim Health Care, Boston and Massachusetts |

**Table 2: Characteristics of included studies (Continued)**

| Study (First author, year(s), country) | Aim | Number of long-term conditions at recruitment | Data collection (IV, FG, PO) | Qualitative methodological approach | Sample (type of long-term condition (number), size, age, sex, ethnicity, location, deprivation) |
| --- | --- | --- | --- | --- | --- |
| Fried, 2008, United States | To examine the ways in which older persons with multiple conditions think about potentially  competing outcomes in order to gain insight into how  processes to elicit values regarding these outcomes can be  grounded in the patient’s perspective. | - At least 5 conditions (based on number of medication taken daily) | FG | Constant comparative method | - Hypertension (88), diabetes mellitus (38), ischemic heart disease (52), congestive heart failure (11), chronic lung disease (24), depression (24), arthritis (73), falls (14), urinary incontinence (21), osteoporosis (20); median number of medications (5), range (3-8); - 66 Patients, mean age 75 years, 67% female; - 76% White; - Community setting: participants’ recruited from senior centers, physicians’ practices, and at a congregate housing site |

**Table 2: Characteristics of included studies (Continued)**

| Study (First author, year(s), country) | Aim | Number of long-term conditions at recruitment | Data collection (IV, FG, PO) | Qualitative methodological approach | Sample (type of long-term condition (number), size, age, sex, ethnicity, location, deprivation) |
| --- | --- | --- | --- | --- | --- |
| O’Brien, 2014, Scotland | To investigate the relationship  between the management of multimorbidity and ‘everyday life work’ in patients living in areas of high socioeconomic  deprivation in Scotland, as part of a programme of work on multimorbidity and deprivation | At least 2 conditions: a range of combinations of problems  (e.g., conditions with or without identified mental health problems; with or without social problems). | Face-to-face semi-structured IV | Continuous, iterative, grounded theory | - Depression (10), diabetes (8), obesity (3), osteoarthritis (2), chronic pain (3); asthma (3), liver disease (3), arthritis (2),   hypertension (4), stress (2), erectile dysfunction (2), chronic obstructive airways disease , alcoholism, neuropathy, colostomy, urostomy, chronic bowel disease, underactive thyroid, anxiety, bronchitis, kidney stones, underactive thyroid, sleep apnoeacerebral vascular accident, blood pressure, stomach ulcer, epilepsy, psoriasis, ischaemic heart disease, stomach problems, mobility (spine problems), stomach problems Crohn’s disease, joint pain;   - 14 Patients, 44-64 years, 6 men; - Patients living in areas of high deprivation (practices had a high percentage of patients in the top 15% most deprived areas in Scotland |
