# Supplementary material for: Living with complexity; marshalling resources: a systematic review and qualitative meta-synthesis of lived experience of mental and physical multimorbidity
Source: BMC Fam Pract. 2015 Nov 24;16:171. doi: 10.1186/s12875-015-0345-3 (PMC4657350; doi:10.1186/s12875-015-0345-3)
Supplement: Additional file 4: Table S3. — Translational table of findings about encounters with complexity. (DOCX 16 kb) [file 12875_2015_345_MOESM4_ESM.docx]

**Additional file 4:Table S3: Translational table of findings about encounters with complexity**

| Descriptor | First order data | Second order themes |
| --- | --- | --- |
|  |  | Impairment, functional loss, social and emotional impact |
| Mobility and functional loss | “I have to try and get through my day in one piece… just piecing the day together takes all my time and energy so I haven’t got anything to give to anything else….” (Coventry 2014); “If it’s not one thing it’s another. I wake up and say ‘What is it today?’ ... It restricts me terrible ... I feel like my life is coming to an end with emphysema and I know it shouldn’t (Townsend et al. 2006)”; “I cannot raise my hands above my head. . .. It (pain) makes me feel so sick. . . it’s in both arms. . . I have to even get my 82 year old mum to wash my hair. . . because of the restriction with my breathing and my cramps.. . .” (Townsend, 2011); “…I have arthritis in my joints and in my hips. I can’t lay on my back or my stomach in bed. I lay on my side so I’m flip-flopping back and forth…I have also got high blood pressure and I take a vertigo pill and just a half a dozen things…but what I’m really concerned about is this chronic fatigue…(Clark & Bennett 2013); “…I can’t walk [and now my daughter] goes to the grocery store for me. I miss going to the grocery store . . . I like to cook, [but] I can’t stand up too long. It’s just when I get up, that’s what irritates me, when I get up and try to walk around…(Sells et al. 2009)”; “[I’m worried] that I might not be able to do anything here anymore, that’s . . .what I fear most… (Loffler et al. 2012)”; “. . .it’s going back to the role of being a breadwinner I would say. . .money was getting desperate, things were getting really bad and I kept blaming myself cos I couldn’t do it and I felt absolutely useless, I couldn’t do nothing to help out. . .(Simmonds et al. 2013)”; “Sometimes I forget [my medicines] and I think, ‘Well, did I take that today?’ I have to sit and think if I took that or not, and then you’re afraid to take it”; “And then, like some days, I don’t want to see anybody. I don’t want to talk to anybody. It’s one of those days where I feel like the world’s against me, and I just want to die. . . . [On those days I] lay in the bed and shut the door. I don’t see nobody” (Schoenberg, 2011); | Bodily and emotional contingencies: temporal and spatial compression (Coventry, 2014); Managing symptoms: the ongoing process of managing symptoms (Townsend et al. 2006); Habitus, capitals and the ill body (Townsend 2011); Experiencing Limitations (Corser, 2011); My body is breaking down like an old car: the physical realities of illness (Clark & Bennet, 2013); Loss (Sells 2009); Coping at emotional level (Loffler et al. 2012); No longer a breadwinner (Simmonds et al. 2013); Multifaceted challenges of MM - more than the sum of its parts; The role of community context and cultural values in self-management (Schoenberg, 2011). |
| Social impact | “[My area] is a place where they send you to die really. They send you there to forget about you…We’re shoved up here with nae [no] facilities…” (O’Brien, 2014); “I just seem to go to bed, sleep, get up, watch the television and vegetate. And I know I shouldn’t be doing that”; “I’m struggling with the stairs. When I come down in the morning I dread having to go back up to the toilet, I dread it”; “I’m not as active as I was. I don’t really go out anywhere. . . . I can’t like walk from here to the bus stop. . . either I wouldn’t manage it, or if it was a good day, the time I would get to the bus stop I would be too tired to go anywhere... So it’s taxi, which is £3 down into Paisley and £3 back up again. So, the money side of it holds me back as well. . . I’m stuck . . . I just feel my whole life is turned totally turned upside down. . . . I would like to have more freedom. . . One money wise and two with my illness. . . (Townsend, 2006; 2008; 2011); “Without places like this clinic here . . . that’s been a godsend for people like me. Up there in Ohio, I couldn’t get no help; no help whatsoever, and that’s another reason why we come down . . . here, because we know that down here in Kentucky, they take care of their people” (Schoenberg, 2011); “I do feel unduly restricted. The truth of the matter is, I get a little impatient sometimes because I can’t go out. I can’t go where I want to go, when I want to go – even the short distances.” (Clarke & Bennet, 2013); “Lately, I’ve been in a lot of pain . . . I put on a lot of weight because I didn’t want to go out walking. It’s really a bad effect . . . It’s been hurting my social life and my life with my wife is very difficult . . .(Sells et al. 2009).” | Difficulties keeping going with everyday life work (in deprived area: O’Brien, 2014); Managing symptoms: the ongoing process of managing symptoms levels of illness: symptoms and conditions; Habitus, capitals and the ill body (Townsend 2006; 2008; 2011); Multifaceted challenges of MM - more than the sum of its parts (Schoenberg, 2011); Habitus, capitals and the ill body (Townsend 2011); Habitus, capitals and the ill body (Townsend 2011); the role of community context and cultural values in self-management (Schoenberg, 2011); I wish I could be more active: the social consequences of multiple chronic conditions (Clarke & Bennet, 2013); Loss (Sells et al. 2009). |
| Emotional impact | “…it’s really difficult because of all the things that are going on around me I can’t really control…and I can’t plan anymore…everything is a jumble, and I lose track of where I am up to” (Coventry, 2014); “ I usually have a lot of doctors’ appointments. That’s mostly what I do, [and] then I have physical therapy . . . It’s kind of depressing because most of my time is spent at the doctors (Sells et al. 2009).” “When you’re used to working and then something just hits you all of a sudden, and you can’t do it no more, it’s like your life is over”  “I worry about the diabetes more than I do anything when they start talking about how you lose your toe first and then your foot and then your leg. That’s what I worry about; that [diabetes] more than every one of them. But I worry about blood pressure, too. They said it could cause a stroke or a heart attack and stuff, and put you in a wheelchair the rest of your life. If you have a bad stroke, it might take you out. I worry about all of them You’ll worry about all of them some time or another; it’ll be on your mind. It’s hard not to.”(Schoenberg, 2011); “If I could get over it and get back to my work ‘cos I’ve always been a provider. . . it gives you a wee bit of self esteem. . . I‘ve never been off in my life and I‘m 50 year old . . . I don’t like being off. . . I still like ma work. . . . Sometimes I can get dead weepy. . . not crying but. . . myself inside as though I need a good cry. . . Well. . . I was always going out in the lorry, I was jumping about, I was fit. . . had my wee moments but I was always good-natured and everything was just flowing along fine and then this thing. And now I’m restricted to the house. . . it’s just terrible” (Townsend, 2011); | Liminal time: between uncertainty and safety (Coventry, 2014); Loss (Sells et al. 2009); the role of community context and cultural values in self-management; multifaceted challenges of MM - more than the sum of its parts (Schoenberg, 2011); Habitus, capitals and the ill body (Townsend 2011); |
