# Supplementary material for: Living with complexity; marshalling resources: a systematic review and qualitative meta-synthesis of lived experience of mental and physical multimorbidity
Source: BMC Fam Pract. 2015 Nov 24;16:171. doi: 10.1186/s12875-015-0345-3 (PMC4657350; doi:10.1186/s12875-015-0345-3)
Supplement: Additional file 5: TableS4. — Translational table of findings about marshalling medicines, relations and emotions. (DOCX 24 kb) [file 12875_2015_345_MOESM5_ESM.docx]

**Additional file 5:TableS 4: Translational table of findings about marshalling medicines, relations and emotions**

| Descriptor | First order data | Second order themes |
| --- | --- | --- |
|  |  | Strategies, behaviours, medicines taking to cope with multimorbidity |
| Self help strategies | “Yes. What I do is, I er, get up, I got up at six o'clock in the morning and have my breakfast, and I got my nebuliser, and then I go out and I got to Piccadilly Station, buy the paper, read the paper, and when it's nearly time for the train I get my ticket go on the train, get off at Southport, have a walk and then maybe sit down for a couple minutes… Yes. Then after that, I usually get the bus back again and get the train home. Yes, yes, and I feel, I feel good, it's funny isn't it? Yes. It is a marvellous feeling it is” (Coventry, 2014); “I make dinner, yes. I do everything. Yes, I go grocery shopping . . . Every place I have to go, I go. Easy. Because I didn’t know the city bus . . . I thought you couldn’t get on the city bus if you’re in a wheel chair, but now they got the facility, they got two big spaces and if you are in a wheelchair, they don’t let nobody go first. That’s it (Sells et al. 2009)”; “Mostly…just walk. That's what my doctor tells me all the time. Walk or ride on a bicycle… She told me to get plenty exercise and I do. She said it really strengthens the muscle around your heart to do that. I’ll sit down some but I don’t never get to the point that I'm huffing and blowing. Go home and take a hot shower and sometimes I rub alcohol on my knees and I'm ready for another day”; “Cause see I've one of those little… pill boxes and they had the days and then all I have to do I can do either one I can record it, I can start on the day and put all my medication in each day and that way I'm certain that I've taken all of it” (Leach, 2008); “Yes, I get lightheaded, well I don’t try to keep on walking, wherever I’m at the sign can say “no loitering,” but I’m going to sit down. They’ll just have to come and move me. But I will stop and I’ll wait until, you know, the feeling has left. Then I’ll start on my merry way, and if it gets too bad I’ll go back home”; “My wife is from Jamaica and I’m from Haiti, and we used to cook with a lot of spices. But, uh, the doctor told me no salt, no hot sauce. My wife is trying to find a way to still cook with a little bit of . . . yes, flavour”; “Well, I don’t have a major health problem. I mean, I have, what to me, they’re annoyances”; I have an 87-year-old aunt that I take care of. Now she is in a nursing home, but before she went to the nursing home I was literally the hunchback of Notre Dame, going from my house to her house . . . doing her meals and her eye treatments. . . . Even now there are days when I feel, oh God, I can’t go, but I get myself ready and I go see about her. And I’m fine, I get there, and I don’t even want to come home”; “I am taking my free medicine, that’s what I call it. I walk every day except Saturday and Sunday. . . . I practice yoga everyday. . . . I stand on my head everyday” (Loeb, 2006); “I am learning to control fatigue, if I don’t control the fatigue, if I don’t watch what I eat…don’t sleep, then I end up in the hospital.” (Corser & Dontje, 2011); “And so I have my alarm set to remind me to take that one [medication] […] because I’ll forget. I’ll get up and be like, okay, I gotta do this, this morning and I’ll forget. Yeah, so I set an alarm so I’ll always remember”; “The time that I’m happiest is when I’m in the woods by myself because I don’t think about diabetes; I don’t think about anything that is stressful; it is just so peaceful and that is like the time when I’m not bothered by anything (Ridgeway et al. 2014)”; “If you try to treat the blood pressure, then the sugar will go up, because what you eat with the blood pressure is not always good on the sugar” (Schoenberg, 2011); “When you’re sitting here all day long, day in day out, and there’s nothing different…that’s why go get depressed…But if you you’ve got something to do then you don’t think of that and the time goes quicker” (O’Brien); “You pace yourself through the day, you know ... If I’m hoovering, I have to shift the settee and what have you. I’ll maybe be doing half the floor and sit down, have a rest, have a cigarette, shift the settee and get up and do the other bit. You know, sit and move the furniture and do the rest of it”; “And then you kind of force yourself, otherwise, I think I would be tempted to, you know, just take codeine and go to bed. It’s that sort of fine line, I’ll have a wee lie down for 5 or 10 minutes and then I’ll get up and go, no matter what” “I figure I manage it quite well. It doesn’t stop me doing much, anything really that I want to do . . .. If your back’s affecting you take your painkillers . . .. When I do get sore I can come in . . . (taxi driving is) probably the only job I could do . . . Aye, I’ve got constant back and neck pain. It’s controllable .. . . But I know if I do something manual I’ll get a sorer back, it’s always there. But I take a lot of quite strong painkillers every day now . . .. I use special things too, like a chair”  (Townsend et al. 2006, 2008); “I read a lot of magazines and newspapers and quite often they include reports about migraine and I know 100% certain how I need to react.”; “I read a lot about my sickness in books (Loffler et al. 2012).” “I’m taking an herb now . . . it comes from the Bible, and it’s called Prophet A, and it’s in the Bible, and you see it in Genesis all the way through Ezekiel. It helped people way back then when they were sick, worn, and all that and it made them well” (Loeb, 2006). | Temporal and spatial customisation: living with multimorbidity (Coventry, 2014); Adaptation (Sells et al. 2009); Modifying dietary intake, exercising; self-care: medicine; Health vigilance (Leach 2008);  coping strategies: self-monitoring (‘body listening’); coping strategies: changing dietary patterns; coping strategies: relying on God (Loeb, 2006); Prioritizing Symptoms/Conditions/Behaviours (Corser & Dontje, 2011); Routinizing self-care; Focus on other life priorities (Ridgeway et al. 2014); Multifaceted challenges of MM - more than the sum of its parts (Schoenberg, 2011); The importance of everyday tasks to manage emotional distress (O’Brien, 2014); Managing symptoms: daily life Managing and maintaining valuable social roles: careful monitoring; Control of symptoms and self-management techniques (Townsend et al. 2006; 2008); Coping at practical level: keep disease under control (Loffler et al. 2012); Coping strategies: relying on God (Loeb, 2006). |
| Decision-making about medicine taking | “I run out of the Zocor [simvastatin] because picking up the eye medication was more important to me, so I got that…So that’s how I played it…I pick up the one that’s most important…” (Elliot, 2007); “I continued with the homeopathic remedies ... I go swimming, and for a sauna, I have to pay membership, but it makes me feel so relaxed, anything that you enjoy helps, I would not like to give that up”; “I ... didn’t accept it [being prescribed antidepressants] before ... Because I always felt it was my fault, ‘I’m not a very nice person’ ... if you’re suffering from diabetes, you need to go and get your insulin ... if you can’t see properly, you need glasses. If you’ve got a chemical imbalance, you need antidepressants. And it was like, ‘Yes, yes, this is it.’ It was just like a revelation”; “I mean, if my colitis is fine I just take my inhalers [for asthma]. I’ve got to take the blood pressure tablets, the ulcer tablets, and the inhalers every day, and the water tablets” (Townsend 2003, 2006, 2008); “This is a good hospital system because I have all of my doctors all in one network, so that makes it easier (Ridgeway et al. 2014)”; “. . I lie in bed and I have an attempt at meditation, she [mental health professional] taught me and I have to do it now before I go to sleep I have 10 minutes meditating … It made me think of good things, it made me think of life, it got me out of thinking of the bad things and I still do it now”; “I would like to be able to open myself to one person  who understands me, my mentality, my way of thinking and then they can give me advice, I mean, we need communication, without we can do nothing”; “No, not so much you are burdening, they have heard it all before, you are there for 15 minutes and you are out the door with a pill” (Simmonds et al 2013);“The doctor says “I want to see you in 3 weeks,” then you have to question the doctor. “Well why do you want to see me in 3 weeks?” Cause now my doctor does not accept Medicare, which means that I have to pay . . . if he prescribes a medication for you now you have to say, “now wait a minute. Don’t you have any samples, because how do I know that I can take this medication?” You’ve gotta learn to do this because of your economic circumstances kind of force you to question your doctor a little bit” (Loeb, 2006); “Yeah, she [her GP] reckoned I'd feel better if I increased the dosage. But I'd rather suffer two or three days – then I've got it under control again” (Loffler et al. 2012); “I’m lucky that they found a cure for it (hypertension) because it’s minor now. It’s under control… I have confidence in the medicine taking control of it… I watch it carefully and have it (blood pressure) taken as often as I can; “I’m lucky that they found a cure for it (hypertension) because it’s minor now. It’s under control… I have confidence in the medicine taking control of it… I watch it carefully and have it (blood pressure) taken as often as I can (Leach 2008); “I said when I retired I wasn’t going to have a schedule. And now I have to get up. I have to wake up at six-thirty to take two pills… I can’t eat until an hour later…” (Noel, 2005); “I was telling somebody earlier I should be taking pain pills for my back, am in serious pain right now, but am taking so many other pills, I said, that unless I am really, really, really really in pain, I don’t take the pain pill”; “I don’t want that third pill . . . the third pill might be the killer, you know what I mean?” (Mishra, 2011); “Well, you have to keep up with what time you have to take this medicine and that medicine, and sometimes they react against each other, so you have to take them at different times”; “[A 62-year-old woman with high blood pressure, arthritis, cancer, and diabetes explained she gave more attention to her diabetes because] I’ve had too many in the family with diabetes, and my dad was a diabetic, and it contributed to his death. And my aunt, she was real close, and she had it really, really bad (Schoenberg, 2011); “…I take my tablets that I have for my blood pressure, my diabetes and my cholesterol and then the aspirin, I forget about the others. I think to myself, I’m knocking about, feeling alright, why bother?”(Bower, 2012); “I take Tylenol for arthritis…in my fingers…but I don’t like to take too many. I take enough pills. I don’t want to have [prescription] painkillers for it…I’m going to pick up the paraffin was today and put the paraffin wax on my hands” (Clark & Bennett 2012). | Future and historical medicine choices (Elliot, 2007; Regular regimen v flexible regimen Reluctance to take drugs v inability to be ‘free of drugs’; being normal and favoured self-image ; Perceived suitability and other treatments and resourses (Townsend 2003, 2006, 2008); Positive aspects of healthcare including systemic and individual provider aspects (Ridgeway et al. 2014); Self-help strategies: Meditation and Yoga; Interventions: helpful including talking therapies, supervised exercise; unhelpful interventions/ antidepressants (Simmonds et al. 2013); Coping strategies: self-advocacy (Loeb, 2006); Coping at practical level (Loffler et al. 2012); Self-care: medicine (Leach, 2008); coordination of taking multiple medications (Noel, 2005); reaching one’s threshold for medication adherence; taking less medication (Mishra, 2011); multifaceted challenges of MM - more than the sum of its parts; Priority; strategies of MM self-management (Schoenberg, 2011); Illness representations specific to multimorbidity (Bower, 2012); I just try to manage things myself: self-care to mitigate physical symptoms and suffering (Clark & Bennett, 2012). |
| Social comparison | “I try to hang out with people who are positive and uplifting. It sounds terrible but I just have nothing to do with two friends I’ve known all my life because all they do is moan. We’ve all got problems. I don’t want to hear about them…” (Clark & Bennett, 2012); “However, you suddenly become very calm and very serene. I felt instinctively that, if this isn’t fixed, right, you’ll die, you know? And then I also thought, well, this guy that they nailed to the cross, right – I’ll lay my fate in his hands. There’s nothing more you can do then – nobody can (Loffler et al. 2012)”; “…trying not to let that erosion take away what you’re about, because people can lose respect for you too if you’re always like sickly”; “And there is even a guy who climbed Mt Everest with type 1 diabetes. So it is not like it keeps you from doing anything (Ridgeway et al. 2014)”; “It's just like having a job really… you have to learn to live with. It's the way I look at it. There’s so many people. I’m not the only one with it… I feel like it's just a normal thing that in life you deal with what you've got and make the best of it and don’t go crying around about it. [laughs] Just deal with it and go on with it”; “But I know I can be worser or Because some people are worse off than I am”; “Oh yeah if you don’t keep it under control it can affect you… to me I think it’s where a lot of people have strokes is because they’re not taking care of whatever condition they have. I have high blood pressure which can cause strokes if you’re not under the doctor and if you know that, then you need to take care of it. But if you don’t know, you can’t take care of something…” (Leach 2008); *“*I feel more relaxed in the body and so […] when I talk with my relatives! And so you may get a better sleep” (Grundberg, 2014); “Isn’t it nice when you have other people and you do it together and help one another, instead of you sitting there thinking how to do it yourself?” (Loeb, 2006); “I couldnae go out myself. . . Sometimes I say: Right I’m doing it’’ And I get so far and I have to turn back. There is nobody in this country that has tried more than me. I have went to hypnosis, I have went everywhere and I would just love to be normal. . . Got a friend that just sits in the house. . . That’s the difference between the two of us. She chooses to sit and watch the TV.. . . I don’t want to sound terrible or judgmental but... She can go out and she doesn’t. . . to me that’s not right. . . (Townsend, 2011). | Go with the flow: learning to live with chronic illness (Clarke & Bennet, 2013); positive approach to life (Loffler et al. 2012); Maintaining a positive attitude; social comparisons (Ridgeway et al. 2014); Normalizing; Striving for control (Leach 2008); ‘perceived and well-managed as a unique individual’: accessibility for dialogue; meeting a competent person; social support (Grundberg, 2014); coping strategies: changing dietary patterns (Loeb, 2006); relational positioning (Townsend, 2011). |
| Social support | “That’s one thing about my kids that I always tell everybody. They help me a lot. If it wasn’t for them, I don’t think I’d be sitting here right now. They cook, clean. They give me my medication. They do everything. In that part, I’m very blessed and satisfied (Sells et al. 2009)”; “Every day, we've got our work, right? We know exactly what we need to do. But what we also know is that we're done by lunch, right? We get up at 7.30/ 7.15 that's when we get up. We have breakfast at eight. My husband prepares breakfast every morning. That's the time I get ready. So, it's 8.30 by the time we're ready. But then we work all morning until lunchtime. Then cooking lunch needs to be done. And when we're finished cleaning the kitchen it's already 1 o'clock. Then we call it a day and take our break (Loffler et al. 2012); “I don’t know, I just try and get on with it and I phone my pal and I blether. If she knows I am down in the dumps she’ll phone me and she’ll phone me back an hour later. She always comes up with something funny, you know, to take your mind off things (Townsend et al. 2006); “I got one sister that I can call like in the middle of the night […]. So sometimes, I have to ask – can I come over? Or can you come pick me up? Or can you just talk to me? [...] I’m feeling this way, I’m kind of depressed, or what should I do about this?”; ““Look at the whole picture together with mind, body, and spirit. [...] So I do spend time in prayer and meditation, which also contributes to my well-being”; “The last time the heart went bad. […] I just walked into [my supervisor’s] office and I said, “Hey, would you have one of the guys walk me to the bus.” So he walked me to the shuttle bus; he rode with me to [the hospital], pushed me in a wheelchair to the emergency room (Ridgeway et al. 2014); “Mornings and evenings when I go to bed. I keep a prayer on my tongue…but basically I do when I get up in the mornings… wake up and say thank you, Lord for another day but actually to pray. I always say a prayer before I do my reading of the Bible then at night” (Leach 2008); “I like to be strong and I’ve also took care of myself. I’ve never asked naebody [nobody] for help. Ever. I help everybody else. But I never ask of for help…Now I just don’t want to ask for help. I just go without. I would just stay in”; “We don’t have much in the mountains, but we have kin. We always have had kin. Plenty of kin around. You look in this hollow and in town and you’ll hear the same family names. If you’re a [common Appalachia surname] you’re probably kin to me.  And if you’re kin to me, you’re probably going to wind up helping me get somewhere, buy those pills, you know” (Schoenberg, 2011). | Social support (Sells et al. 2009); Coping at social level: can do approach to life; Coping at emotional level: positive approach to life (Loffler et al. 2012); Enlisting support from others (Ridgeway et al. 2014); Accepting help to support everyday life work (O’Brien, 2014); strategies of MM self-management (Schoenberg, 2011). |
