# Supplementary material for: Living with complexity; marshalling resources: a systematic review and qualitative meta-synthesis of lived experience of mental and physical multimorbidity
Source: BMC Fam Pract. 2015 Nov 24;16:171. doi: 10.1186/s12875-015-0345-3 (PMC4657350; doi:10.1186/s12875-015-0345-3)
Supplement: Additional file 6: Table S5. — Translational table of findings about (self)-preservation and prevention. (DOCX 17 kb) [file 12875_2015_345_MOESM6_ESM.docx]

**Additional file 6:Table S5. Translational table of findings about (self)-preservation and prevention**

| Descriptor | First order data | Second order themes |
| --- | --- | --- |
|  |  | Motivations, decision making and meaning of coping strategies |
| Understanding motivations to make decisions to self manage | “You’ve only got yourself and you have to self-discipline yourself. Yes, I must have three meals a day. Yes, I have to have salad every day and I have to have my oatmeal…You’re your own policeman”; “There’s no need for me to see my doctor. There’s nothing she can do – unless I broke my leg or something stupid like that…There’s nothing she can do. My spine is extremely painful but there’s nothing they can do with that…I just have to sit it out” (Clarke & Bennet, 2013); “And if I don’t like what the doctor is telling me, and me and them ain’t on the same boat, use your common sense. Get rid of them and get another one. […] You got to be on the same page”; “It’s just things that I have to do, and I know I have to do them if I’m going to get well. There’s really no problem about it. I just do it because I know that’s what I’m supposed to do”; “It all goes back to paying attention to your body and being the captain of your own ship. If somebody tells you something, don’t blindly do it. Make sure that you know (Ridgeway et al. 2014)”; “When I was working, I travelled a lot and being on the road – diet, exercise, everything was gone. See if you’re gone sometimes three weeks out of the month, you’ve blown it. And I don’t think the medical advice or care we get takes into consideration some of the demands of the job that we do” (Noel, 2005); “Nobody knows your body better than you. . . You got to be strong, and the best way to take care of your disability [is by] doing the best you can for yourself, you can’t depend on doctors — they are only human. You got to work with them” (Mishra, 2011); “Well, normally, I just try and sort of talk to meself and say ‘come on you’ve got to start doing something’ and you know, normally the mood will lift” (Simmonds et al. 2013); “I have battled on and I end up. . . in bed for weeks. So it’s trying to get a balance between doing enough that satisfies me, that I’m not relying on everyone and I’ve  still got independence, and cutting off, so that I know I won’t make myself any worse. . . I find it so hard to strike the balance. . . Sometimes I say: ‘Bugger it. I’m just going to go ahead and do it.’ It’s a case of me fighting the disease. I know it’s silly and I’m only hurting myself but I just think I’ve always been a fighter so my way of fighting it is like get on and do it and I think to myself: ‘You’re going to pay for this’ [laughs]”; “I couldn’t bear watching my three young children coming in every day and seeing me in bed. I had to do something, I knew I had to do something, and I was very loathe to take these antidepressants”; “…I don’t want to think along those lines. I’m too young. I mean it’s ridiculous. Fifty-two, you know, at 52, to think of stair lifts and toilets downstairs, do you know what I mean?” (Townsend, 2011, 2003, 2006); “I think I would go back to the thing that I fear the most, being incapacitated and living, so I would choose whatever would prevent that” (Fried, 2008); “I don’t want to be like my father who died with amputations” (Loeb, 2006). | The doctor can only do so much; the rest is up to you: self-care in response to the limits of physician directed care; You control your health: self-care as a moral imperative (Clarke & Bennett, 2013); Being proactive with providers; Adaptation and normalising of self-care; Preserving autonomy and independence (Ridgeway et al. 2014); Self-management learning needs (Noel, 2005); Lack of shared information and decision making (Mishra, 2011); Self-talk and thinking processes (Simmonds et al. 2013); Habitus, capitals and the ill body; Drugs as facilitators to perform social roles v drugs and inability to perform social roles; Being normal and favoured self-image (Townsend 2011, 2003, 2006); Shifting from disease-specific to global, cross-disease outcomes (Fried, 2008). |
| Behavioural and social coping strategies | “You don’t feel happy about your health problems but there’s nothing much you can do about it except for try to accommodate to it…and you cannot let your health problems take over your life” (Clark & Bennett, 2013); “I do have a financial advisor and then I have been talking to friends that are either retired or nearing retirement to find out what they are going to do and just trying to get ideas (Ridgeway et al. 2014); “ Old folks deal with everything that they get, as best they can. . . . You feel that you are fortunate to have lived a [long life] . . . although our situations are bad, although I’m never going to get another pair of legs, or I guess I’m going to have hypertension for the rest of my life, or arthritis, or glaucoma, or something else that I picked up . . . I am just going to have to live with it. And that’s the way we seniors do for the most part”; “He [doctor] said “you have to have surgery.” I said, “I’m not having surgery.” . . . I just went to church and I mean those people prayed for me and my back, those herniated discs, I don’t even know. I have herniated discs there, nothing else was done, my back is totally healed . . . *totally* [italics added]. . . . Now I can do *anything* [italics added]; “When you get your leg amputated, you have to learn all over again how to walk, how to go up the stairs, and sometimes that can be pretty difficult. . . . I took a little part-time job as a bartender. You know as a bartender, you go psst, psst, psst [indicating he was running back and forth]. I don’t drink or anything like that. I did that [took the job] to keep myself exercising in the leg itself, and it worked miracles for me”; “I like when I come here [senior center], I find more activities to be doing… having something to look forward to” (Loeb, 2006); “I found that people really don’t want to know. . . my friend’s do but not all the time, ma sons do but not all the time and it’s a very true saying: ‘‘Laugh and the world laughs with you, cry and you cry alone’’’. . . So I tend to over compensate with makeup, with getting probably overdressed, big cheesy grin on the face, laugh a minute and inside you’re crying and then you come home and you take it all off and you cry. . . again that’s me losing part of my independence. . . you don’t want to be talking to people. . . if you’re in pain every day. . . they would all just say: ‘‘Och no she’s just moaning’’; “People ... they say that I work too much ... but it’s not like that at all, it’s completely different from that. If I did not have that [work] what, what would I be, just sitting here and that’s it (Townsend, 2011; 2006); “And that proves once again that you need to listen to your own body and decide all for yourself. What is good for me? And if I didn’t do that, no doctor would be able to help me (Loffler et al. 2012); “I’ve got to be doing something. I’ve got to be tidying up. I’ve got to make sure my house is the way I want. No I wouldn’t sit in my own filth, no. I’ve got to do my housework whether it kills me or not” (O’Brien, 2014). | Go with the flow: learning to live with chronic illness (Clark & Bennett 2013); Planning for the future (Ridgeway et al. 2014) coping strategies: dealing with it; relying on God; exercising; engaging in life (Loeb, 2006); illness and symbolic violence; managing and maintaining valued social roles: being a worker and mum (Townsend, 2011, 2006); Coping at practical level: keep disease under control (Loffler et al. 2012); The symbolic significance of everyday work (O’Brien, 2014). |
